# Vaccination with recombinant NetB toxin partially protects broiler chickens from necrotic enteritis

**DOI:** 10.1186/1297-9716-44-54

**Published:** 2013-07-16

**Authors:** Anthony L Keyburn, Ricardo W Portela, Kathy Sproat, Mark E Ford, Trudi L Bannam, Xuxia Yan, Julian I Rood, Robert J Moore

**Affiliations:** 1 CSIRO Biosecurity Flagship, Australian Animal Health Laboratory, Geelong 3220, Australia; 2 ARC Centre of Excellence in Structural and Functional Microbial Genomics, Department of Microbiology, Monash University, Clayton 3800, Australia; 3 Poultry Cooperative Research Centre, Armidale, Australia; 4 Health Sciences Institute, Federal University of Bahia, Salvador, Brazil

## Abstract

NetB toxin from *Clostridium perfringens* is a major virulence factor in necrotic enteritis in poultry. In this study the efficacy of NetB as a vaccine antigen to protect chickens from necrotic enteritis was examined. Broiler chickens were immunized subcutaneously with purified recombinant NetB (rNetB), formalin treated bacterin and cell free toxoid with or without rNetB supplementation. Intestinal lesion scores and NetB antibody levels were measured to determine protection after mild oral gavage, moderate in-feed and heavy in-feed challenges with virulent *C. perfringens* isolates. Birds immunized with rNetB were significantly protected against necrotic enteritis when challenged with a mild oral dose of virulent bacteria, but were not protected when a more robust challenge was used. Bacterin and cell free toxoid without rNetB supplementation did not protect birds from moderate and severe in-feed challenge. Only birds immunized with bacterin and cell free toxoid supplemented with rNetB showed significant protection against moderate and severe in-feed challenge, with the later giving the greatest protection. Higher NetB antibody titres were observed in birds immunized with rNetB compared to those vaccinated with bacterin or toxoid, suggesting that the in vitro levels of NetB produced by virulent *C. perfringens* isolates are too low to induce the development of a strong immune response. These results suggest that vaccination with NetB alone may not be sufficient to protect birds from necrotic enteritis in the field, but that in combination with other cellular or cell-free antigens it can significantly protect chickens from disease.

## Introduction

Necrotic enteritis in chickens is a common bacterial disease that costs the global poultry production industry an estimated US$2 billion annually [1]. The causative agent is the bacterium *Clostridium perfringens.* Currently, ionophore anticoccidials or antibiotic growth promoters are used to control necrotic enteritis [2]. However, the risk of antibiotic resistance and consumer pressure has prompted the industry to reduce the use of in-feed antibiotics and it is likely that the use of ionophore anticoccidials will also be reduced. In the European Union, the use of most antibiotic growth promotants has been banned, and necrotic enteritis remains an ongoing issue for producers in these countries [3,4]. This situation has increased the need to develop other methods to control necrotic enteritis in poultry. Vaccination is an alternative approach that could be deployed to manage necrotic enteritis in the absence of antibiotics and anticoccidials.

Vaccines against other clostridial diseases in production animals have been widely and successfully used for many years and are based on protection from specific toxins produced by the bacteria that are associated with the particular disease [5]. Necrotic enteritis in chickens is a notable exception; it is an economically important clostridial disease for which there are limited vaccines available. Although necrotic enteritis has been recognised as a significant clostridial disease of chickens for 50 years [6], progress towards the development of a vaccine has been very limited until recently. Historically, *C. perfringens* alpha-toxin was implicated as the major virulence factor in the disease, which led to vaccine development efforts based around this toxin. Several experimental vaccines based on alpha-toxin have been reported and they have had variable protective success [7-9]. However, an alpha-toxin deficient mutant strain of *C. perfringens* has been shown to retain full virulence [10], indicating that the toxin is not an essential virulence factor. Despite this observation it is clear that antibodies raised against this toxin can partially protect birds from disease. Although alpha-toxin is a secreted protein, Zekarias et al. [9] have shown that some of the protein remains associated with the cell membrane. It is presumably immune interaction with this cell-associated protein that provides the protective effect seen with some alpha-toxin based vaccines. The fact that vaccines using live attenuated alpha-toxin negative strains of *C. perfringens* are effective against avian necrotic enteritis [11] demonstrates that there must be other antigens of *C. perfringens* that are capable of inducing a protective immune response. Some of these protective antigens have been identified in recent studies [12,13].

Recently, a secreted β-pore forming toxin, NetB, has been isolated from a virulent chicken isolate of *C. perfringens* and shown to be essential for disease induction [14]. NetB toxin has been found in most *C. perfringens* isolates from necrotic enteritis-diseased birds, but is uncommon in isolates recovered from healthy birds [15-17]. As an important virulence factor, NetB represents an attractive vaccine candidate, as shown in a recent study where vaccination with NetB induced some protection of broiler birds against experimental necrotic enteritis [18]. The studies reported here not only test whether NetB can be used as a protective vaccine antigen as a single subunit vaccine, but investigate whether NetB in combination with other antigenic proteins, either whole cell bacterin or secreted *C. perfringens* toxoid, can enhance protection in chickens against necrotic enteritis.

## Materials and methods

### Strains

*C. perfringens* strains EHE-NE18 [10] and WER-NE36 were used as challenge strains in the in vivo necrotic enteritis disease induction models described below. EHE-NE18 (Type A, *netB*^+^) has been previously shown [10] to be virulent in an in vivo model while WER-NE36 (Type A, *netB*^+^) was isolated recently from a clinical case and shown to be highly virulent in our disease models.

### Vaccine preparation and delivery

A single EHE-NE18 colony from a Tryptose Sulphite Cycloserine agar plate (TSC agar, Oxoid, Basingstoke, United Kingdom) was inoculated into 20 mL of Trypticase-peptone-glucose (TPG) medium and grown overnight at 37°C. Ten mL of the resultant culture were inoculated into one litre of TPG medium and grown at 37°C to an OD_600nm_ of 0.8 – 1.0. The culture was centrifuged at 6000 *g* for 10 min at 4°C, filtered through a 0.45 μm membrane and concentrated by ultrafiltration through a 10 kDa membrane (Merck Millipore, Massachusetts, USA) to 40 mL (25×). The bacterial cells were resuspended in 40 mL of phosphate buffered saline (PBS; 137 mM NaCl, 10 mM phosphate, 2.7 mM KCl, pH 7.4) and sonicated (three times for 30 s) to disrupt the cellular membranes and release cytoplasmic proteins. Formaldehyde (40%) was added to both the concentrated supernatant and the cells to a final concentration of 0.3% (v/v). Bacterin comprised 50:50 (v/v) bacterial cells and culture supernatant, while toxoid consisted of formaldehyde-treated culture supernatant only.

Recombinant NetB (rNetB) was expressed and purified (98% purity) as previously described [14] and formalin treated as described above. Fifty μg of rNetB per bird was used for NetB subunit, bacterin- and toxoid-supplemented vaccines. Each bird was vaccinated subcutaneously at day 7 and day 17 using CSIRO triple adjuvant [19] (comprising 60% (v/v) Montanide, 40% (v/v) antigen combined with Quil A, 3 mg/mL, and DEAE-dextran, 30 mg/mL in PBS) in a total volume of 500 μL.

### Necrotic enteritis disease induction models

Two different challenge methods were used: a direct oral gavage challenge with small volumes of culture, which may be more akin to challenges that occur in the field, and a large volume in-feed challenge that gives a more robust challenge, resulting in higher levels of disease in a greater percentage of birds. These oral gavage and in-feed necrotic enteritis disease induction models were performed essentially as previously described [10,20], with some modification to the timing to fit in with vaccination schedules. Briefly, groups of 10 chickens were kept in adjacent, but separate, pens in an animal isolation facility. Commercial 1-day-old Ross 308 broiler chickens were fed an antibiotic-free chicken starter diet containing 20% (w/w) protein for 22 days. On the morning of day 23 the feed was changed to a high protein wheat-based feed containing 50% fishmeal. On day 28, chickens were euthanized with inhaled carbon dioxide and their small intestines (duodenum to ileum) examined for gross necrotic lesions. Intestinal lesions in the small intestine were scored as before [10]: 0 = no gross lesions; 1 = thin or friable walls; 2 = focal necrosis or ulceration (1–5 foci); 3 = focal necrosis or ulceration (6–15 foci); 4 = focal necrosis or ulceration (16 or more foci); 5 = patches of necrosis 2–3 cm long; 6 = diffuse necrosis typical of field cases. The statistical significance of differences between groups was assessed using Kruskal-Wallis one-way ANOVA. All animal experiments were assessed, approved and monitored by the Australian Animal Health Laboratory’s Animal Ethics Committee. Experimental models of *C. perfringens* infection to induce necrotic enteritis require the addition of predisposing factors. The most widely published models either use high protein feed or *Eimeria* infection. *Eimeria* infection can cause immunological stress, which may be inappropriate in a vaccine study. Therefore, we chose to use high protein as the predisposing factor in this study.

#### Oral gavage challenge

For the oral gavage challenge, *C. perfringens* strain EHE-NE18 was grown in fluid thioglycollate broth (FTG; Difco) with the addition of 2% (w/v) soluble starch and 1.5% (w/v) thiopeptone and incubated at 37°C for 14 h. On the evening of day 24 the feed was withdrawn and each bird was orally challenged with 1.5 mL of *C. perfringens* culture (10^9^ to 10^10^ CFU). On day 25 birds were again orally challenged and feed contaminated with *C. perfringens* (1:10 (v/w) culture to feed) was administered.

#### In-feed challenge

For the in-feed challenge, 1–2 *C. perfringens* (EHE-NE18 or WER-NE36) colonies grown on TSC agar were transferred into 10 mL of cooked meat medium (CMM; Difco Becton Dickinson, Maryland, USA) and incubated at 37°C for 18 h. One mL of the resulting culture was used to inoculate 20 mL of FTG, and, after incubation at 37°C for 18 h, 1 mL was used to inoculate 20 mL of CMM and incubated at 37°C for 18 h. Twenty mL of the final CMM culture was used as the inoculum for 800 mL of FTG medium, and after 18 h incubation at 37°C, the culture was mixed with feed for the challenge. A separate serially passed culture was prepared for each challenge feeding (*n* = 4). High protein-feed and FTG culture were mixed in a ratio of 3:4 (v/w). The mixture was then placed into feed trays. Birds were fed the culture/feed mix twice a day on days 26 and 27. Trays were cleaned and the remaining feed discarded prior to each subsequent feeding.

### Experimental design

For NetB immunogenic studies two groups of 10 birds were challenged in-feed (described above) with either EHE-NE18 or WER-NE36. Blood samples from the wing (brachial vein) were taken and serum collected directly before challenge and 6 and 10 days post challenge. Serum anti-NetB IgY levels were determined by enzyme-linked immunosorbent assay (ELISA).

The vaccine studies were performed with the same vaccination regime but separated into three different challenge models; (i) oral gavage challenge with EHE-NE18 to produce mild disease, (ii) in-feed challenge using EHE-NE18 to produce moderate levels of disease, and (iii) an in-feed challenge with WER-NE36 to produce the most severe disease. For vaccine studies a blood sample was taken from each bird, immediately following euthanasia, for serum analysis by ELISA and Western blotting.

### Western blotting and immunoassay detection of antibodies

NetB-specific antibody levels were determined by the end-point dilution method using an ELISA. Microtiter plates (Nunc Maxsorp, Roskilde, Denmark) were coated with rNetB protein (1 μg/mL in 0.1 M sodium carbonate buffer, pH 9.6) for 16 h at 4°C. The coated plates were washed twice with PBS (pH 7.6) and blocked for 3 h at room temperature with 5% (w/v) bovine serum albumin (BSA; Sigma Aldrich, Missouri, USA) in PBS (PBSB). The plates were washed twice with PBS and sera from the immunized birds, and the concurrent control groups, diluted 1:400 in 1% BSA, were added and the plates incubated for 1 h at 37°C. The plates were washed 5 times with PBS plus Tween 20 (0.05% (v/v), PBST) and once with PBS before the addition of goat-anti-chicken IgY horseradish peroxidase conjugate (KPL, Maryland, USA) diluted 1:2000 in PBSB and incubated for 1 h at 37°C. After extensive washing of the plates with PBST (5×) and PBS (1×) the colour reaction was developed by using a tetramethylbenzidine alkaline phosphate substrate kit (Invitrogen), following the manufacturer's instructions. The reactions were stopped by the addition of 50 μL of 0.5 M NaOH and the absorbance at 450 nm was measured in a microplate spectrophotometer (BioTek). The specific antibody level of the immune serum was expressed as the *A*_450_ value above the cut-off [21], which was defined as the mean absorbance value of the unimmunized plus three standard deviations.

A recombinant NetB preparation was run on SDS-PAGE (NuPAGE^®^ Novex 4–12% Bis-Tris gel, Invitrogen) in MES SDS buffer (NuPAGE^®^MES SDS Running Buffer, Invitrogen, California, USA). Protein was transferred onto PDVF (Invitrogen) membrane and probed with immune serum from vaccinated birds. Blots were developed with an ECL Western Blotting kit (Amersham Biosciences, GE Life Sciences, Bunkinghamshire, United Kingdom) and the results recorded on autoradiographic film.

## Results

### NetB is immunogenic in chickens

**Figure 1:**
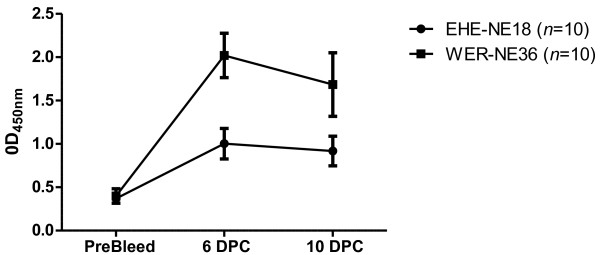
**NetB specific IgY production in birds inoculated with two different *C. perfringens *strains.** Two groups of 10 birds were challenged in-feed (days 26 and 27) either with EHE-NE18 (●) or WER-NE36 (■) and serum collected on days 26 (pre-bleed), 33 (6 days post-challenge, 6 DPC) and 37 (10 days post challenge, 10 DPC). The levels of serum IgY antibodies against NetB were measured by ELISA and expressed as the average optical density at 450nm. The error bars represent SEM.

To investigate whether in vivo produced native NetB was able to invoke an immune response during an infection, groups of 10 birds were challenged separately, in-feed (see material and methods for details), with two different virulent *C. perfringens* strains, EHE-NE18 and WER-NE36, and allowed to recover from the infection. Serum collected at pre-infection, 6 and 10 days post-challenge, was used in a NetB-specific ELISA. Pooled serum from birds challenged with either EHE-NE18 or WER-NE36, showed strong anti-NetB antibodies, with the highest levels observed 6 days post challenge (Figure 1). These results demonstrated that NetB stimulates an immune response in birds that have been challenged with virulent strains.

### rNetB induces a protective immune response

**Figure 2:**
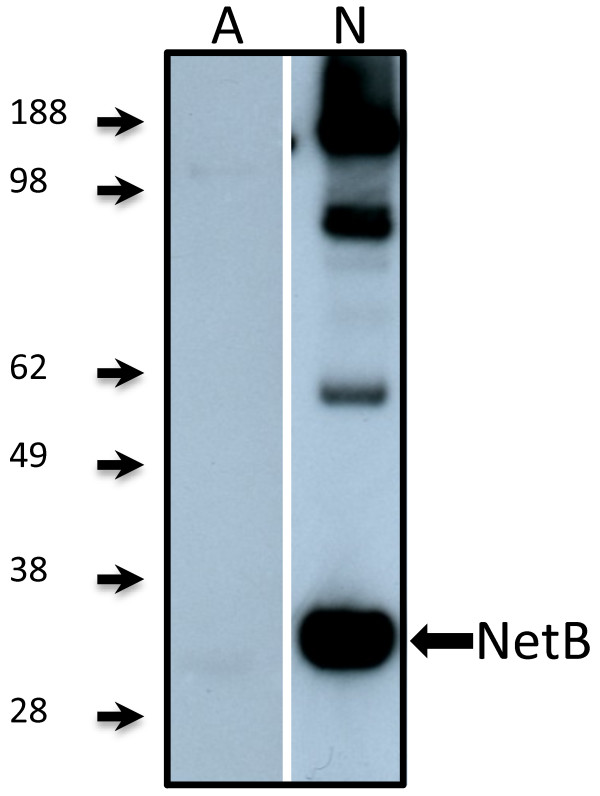
**Western blot of serum from broiler chickens immunized with rNetB.** Recombinant NetB was electrophoresied on a 4%-12% SDS-PAGE and transferred onto a PVDF membrane. The blot was developed with an ECL Western blotting kit and results recorded on autoradiographic film. SeeBlue Plus2 prestained marker (Invitrogen) was used as a size marker (kDa). Birds were immunized with 50 μg of rNetB with CSIRO Triple adjuvant (days 7 and 17) and serum collected on day 28. A, probed with sera from birds vaccinated with adjuvant only; N, probed with sera from birds vaccinated with rNetB in adjuvant.

**Figure 3:**
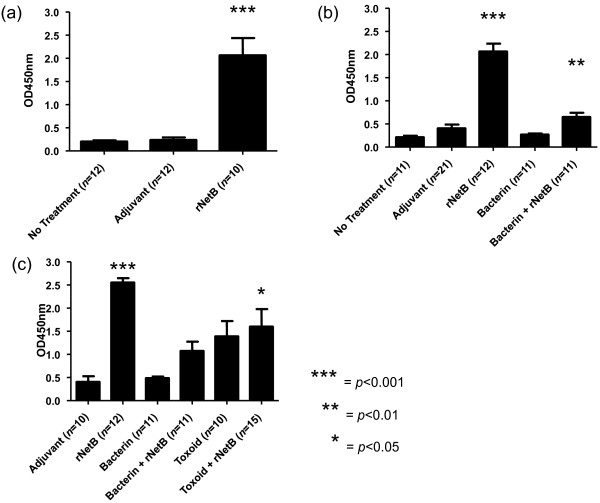
**Serum IgY responses of broiler chickens immunized with clostridial proteins.** Chickens were immunized subcutaneously with the indicated vaccines on days 7 and 17, challenged with *C. perfringens*, and the levels of serum IgY antibodies against NetB were measured by ELISA at day 28. Adjuvant only and no treatment groups were used as controls. **(a)** Birds were immunized and challenged orally with EHE-NE18 (days 24 and 25). **(b)** Birds were immunized and challenged in-feed with EHE-NE18 (days 26 and 27). **(c)** Birds were immunized and challenge in-feed with WER-NE36 (days 26 and 27). The error bars represent SEM.

To determine the immunogenicity of purified rNetB, chickens were immunized at day 7, boosted at day 17, and oral gavage challenged with EHE-NE18 at days 24 and 25. Serum was collected at day 28 post-hatch. These sera were tested for NetB specific antibodies using Western blot analysis (Figure 2) and ELISA (Figure 3a). Chickens immunized with rNetB had significantly higher levels of anti-NetB IgY antibodies (*p* < 0.001) than birds treated with adjuvant alone or the no treatment controls. Western blot analysis using sera from the rNetB vaccinated birds showed strong antibody binding to rNetB (33 kDa).

**Figure 4:**
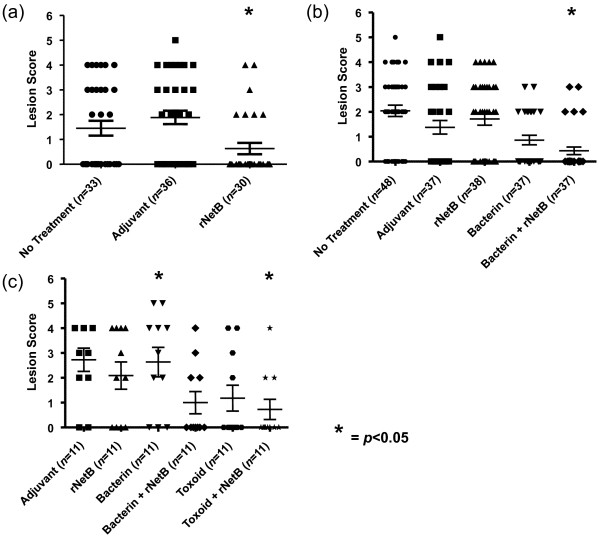
**Lesion scores of broiler chickens immunized with clostridial proteins.** Chickens were immunized subcutaneously with the indicated vaccines on days 7 and 17, challenged with *C. perfringens*, and the lesions scores assessed at day 28. The solid horizontal bars represent the average lesion score in each group. Intestinal lesions in the small intestine (duodenum to ileum) were scored as previously reported: 0, no gross lesions; 1, thin or friable walls; 2, focal necrosis or ulceration (1 to 5 foci); 3, focal necrosis or ulceration (6 to 15 foci); 4, focal necrosis or ulceration (16 or more foci); 5, patches of necrosis 2 to 3 cm long; 6, diffuse necrosis typical of field cases. **(a)** Birds were immunized and challenged orally with EHE-NE18 (days 24 and 25). **(b)** Birds were immunized and challenged in-feed with EHE-NE18 (days 26 and 27). **(c)** Birds were immunized and challenge in-feed with WER-NE36 (days 26 and 27). Error bars represent SEM.

To determine if vaccination with rNetB can protect birds from disease, birds were immunised with rNetB and then challenged with pathogenic *C. perfringens*. Protection was assessed by the average lesion score of the treatment groups. The rNetB vaccine provided statistically significant (*p* < 0.05) protection against an oral gavage *C. perfringens* challenge in comparison to the adjuvant and no treatment control groups (Figure 4a). There was a 58% reduction in the number of birds with lesions and a significant reduction in the average lesion score (65% reduction, *p* < 0.05) when the rNetB-vaccinated group was compared with the combined adjuvant and no treatment groups.

A second, more aggressive in-feed, homologous challenge model was used to further characterise the protective efficacy of the rNetB vaccine. To determine if improved efficacy could be obtained we also immunised other birds with either whole cell bacterin or whole cell bacterin supplemented with rNetB. The birds immunised with rNetB or bacterin alone were not protected against this more aggressive homologous challenge (Figure 4b). However, birds immunised with bacterin supplemented with rNetB had a statistically significant reduction in lesion scores compared to both the negative control groups (*p* < 0.05), with 67% reduction in birds with lesions and 76% reduction in average lesion score. Birds immunised with rNetB alone (Figure 3b) had the highest level of anti-NetB IgY antibodies whereas birds immunised with bacterin had very low levels. Those birds immunised with bacterin supplemented with rNetB had significantly higher (*p* < 0.05) levels of anti-NetB IgY antibodies compared to the no treatment control birds.

To determine if bacterin supplemented with rNetB could protect against a heterologous challenge we immunised birds with rNetB, bacterin and bacterin supplemented with rNetB. We also immunised chickens with toxoid (cell free inactive proteins) and toxoid supplemented with rNetB. The birds were challenged, in-feed, with the most highly virulent strain available to us, WER-NE36. Those birds immunised with either rNetB or bacterin showed no reduction in lesion score compared to the adjuvant control group (Figure 4c). The group immunised with toxoid had a lower average disease score but was not statistically significantly protected (*p* > 0.05). Only chickens immunised with bacterin or toxoid supplemented with rNetB showed statistically significant (*p* < 0.05) decreases in lesion score compared to the adjuvant control group (Figure 4c), with the rNetB-supplemented toxoid vaccine reducing the number of birds with lesions by 67% and the average lesion score by 74%. Birds immunised with rNetB alone had the highest levels of anti-NetB IgY antibodies (Figure 3c). Similar to the previous vaccination trial, chickens immunised with bacterin had very low NetB-specific antibody levels while the group vaccinated with bacterin supplemented with rNetB had higher levels compared to the adjuvant control group. Birds immunised with toxoid or toxoid with rNetB had similar levels of anti-NetB IgY antibody (Figure 3c) although only toxoid with rNetB was significantly higher (*p* < 0.05) compared to the adjuvant control group.

## Discussion

This study has evaluated whether NetB, a major virulence factor in necrotic enteritis, is an effective protective antigen when used as a subunit vaccine or as a supplement to either traditional bacterin or toxoid vaccines. rNetB alone was protective against a mild challenge with a virulent *C. perfringens* strain, but was not sufficient to protect against a heavy in-feed challenge. Immunisation with bacterin or cell free toxoid supplemented with rNetB significantly protected birds against necrotic enteritis following a heavy challenge with a heterologous strain of *C. perfringens* (Figure 4b,c). We conclude that rNetB has considerable potential for the development of vaccines against necrotic enteritis.

Our results are consistent with other studies that have used cell free toxoids as vaccines [22-24]. Saleh et al. [22] used two *C. perfringens* isolates from diseased birds (type A and C) to generate three toxoid vaccines (type A, type C, and type A plus type C). A significant decrease in disease was observed with all three toxoid vaccines, with the highest protection coming from the combined type A and C toxoid. The birds immunised with this toxoid also had higher antibody titres compared to the other toxoid vaccines. Other studies have used toxoids to immunise broiler breeder hens, on the premise that they will develop maternal antibodies that are transferred to the broiler progeny [23,24]. Lovland et al. [24] immunized birds with type A and C cell free toxoid and found that the type C toxoid protected progeny better than type A. For both toxoids, the level of anti-alpha-toxin IgY antibodies was monitored in the hens and in day old progeny. The antibody titres in vaccinated birds were higher than those in unvaccinated hens and the data suggested a protective effect on the progeny against subclinical necrotic enteritis. However, in their study birds immunised with the type C toxoid had lower anti-alpha-toxin IgY antibody levels, but gave the better protective effect. This result may be due to the type C strain also having higher levels of protective antigens. While the NetB status of the strains used to produce the cell-free toxoids in these studies is not known, it is unlikely that the strains that were used produced NetB since both strains were isolated from mammals [24] and there is only one report of a NetB positive *C. perfringens* being isolated from an animal other than poultry [17].

A similar study by Crouch et al. [23] immunised broiler layer hens from Germany and Italy with the commercially available necrotic enteritis vaccine (NetVax™). The safety and efficacy of the *C. perfringens* type A alpha-toxoid was investigated. Again, the NetB status of the strain used to prepare the vaccine was not reported, but it was presumably NetB negative as the strain was isolated from cattle. Vaccination resulted in a significant increase in anti-alpha-toxin antibody in the hens and antibodies were detected in the progeny. In contrast to the previous study [24], which detected *C. perfringens* at greater than 4 × 10^4^ CFU/g, chicks from NetVax™ vaccinated hens had no detectable levels of *C. perfringens*. The absence of *C. perfringens* cells in chicks from vaccinated hens suggests that maternal vaccination with NetVax™ reduces the ability of the bacteria to colonise the gastrointestinal tract of chickens.

In our study, the addition of rNetB to bacterin or toxoid preparations from a NetB-producing *C. perfringens* strain significantly lowered the average lesion score compared to the adjuvant control. Birds immunized with bacterin had low titres of anti-NetB antibody, suggesting that the native NetB protein is in low abundance in these preparations (Figure 3b,c). In vitro grown cultures contain too little NetB to induce a protective immune response, hence supplementation with exogenous recombinant NetB was required to induce a significant anti-NetB immune response.

A recent study investigated the immunoprotective potential of native culture supernatants from disease and non-disease derived *C. perfringens* isolates [25]. Of the eight strains tested, two supernatants provided full and partial protection (strains 23 (NetB^+^) and 48 (NetB^-^), respectively). The authors concluded that neither alpha-toxin nor NetB were solely immunoprotective. Unfortunately, the sera from the vaccinated birds were not analysed to identify the possible protective antigens, including NetB and alpha-toxin. Our results are in agreement with this study; we confirmed that rNetB alone was not highly immunoprotective against moderate *C. perfringens* challenge and demonstrated that supplemented rNetB, in conjunction with other antigens, increased the protective response compared to native antigens. This result is not surprising since *C. perfringens* infections are multifactorial and most clostridial vaccines are made up of a cocktail of protective antigens, most often cell free toxoids, rather than a single subunit based vaccine.

Analysis of the convalescent sera from birds challenged with strains EHE-NE18 and WER-NE36 indicated that NetB IgY antibodies were generated during infection, which is in agreement with a study that looked at both alpha-toxin and NetB antibody levels in birds that have been experimentally challenged and birds from natural outbreaks [26]. That study found that challenged birds had a peak anti-NetB IgY antibody level 7 days post-challenge, after which it dramatically dropped back to unchallenged control levels 14 days post-infection. The results from our work and Lee et al. [26] highlight the importance of NetB as a protective antigen and its significance in disease.

In this study rNetB, when used as a subunit vaccine, was protective against oral gavage *C. perfringens* challenge, but not against a more severe challenge. This result is similar to a recent study where birds vaccinated with rNetB had a significant decrease in lesion score compared to the adjuvant control [18]. These authors investigated the role of cellular immunity in protection against *C. perfringens*/*Eimeria maxima* co-infection and found that vaccination with ISA 71 VG adjuvant plus recombinant *C. perfringens* antigens reduced transcripts of proinflamatory cytokines in intestinal intraepithelial lymphocytes. Other recombinant antigens including alpha-toxin, pyruvate:ferredoxin oxidoreductase, a hypothetical protein, glyceraldehyde-3-phosphate dehydrogenase, fructose 1,6-biphosphate aldolase, endo-β-*N*-acetylglucosaminidase, and phosphoglyceromutase have also been shown to significantly protect birds from necrotic enteritis [7,13,18]. The role of alpha-toxin in immunity has been studied extensively and it has been shown to confer variable levels of protection [7-9,13,23,24,27]. It is clear that there is a growing body of evidence that other antigens, other than alpha-toxin, can confer protection against disease [11-13,26,28]. NetB can be added to the list of protective antigens against necrotic enteritis [18].

The findings in this study represent the first steps towards the development of an effective vaccine against necrotic enteritis in chickens. The vaccination regime used in the experiments is unlikely to be appropriate for widespread use in broiler chicks because of the multiple handling of individual birds required. A more industry appropriate regime would be to vaccinate the breeder hens, such as used in other studies [23,24] to induce protection in progeny chicks; or vaccine delivery in feed, water, or aerosolized, but that would require sophisticated adjuvant and delivery technology.

In conclusion, we have shown that rNetB immunization can significantly protect birds against disease, but not against a severe challenge. The greatest protection observed in this study came from birds immunized with cell free toxoid or bacterin supplemented with rNetB; these “enhanced toxoids” may be suitable candidates for use in the poultry industry in the near future.

## Competing interests

ALK, JIR and RJM have a patent on NetB and its use in vaccines: Clostridial Toxin NetB. US Patent no. 8,263,088 B2.

## Authors’ contributions

ALK, JIR, RJM conceived and designed the experiments. ALK, RWP, KS, MEF, TLB, XY, RJM performed the experiments and analysed the data. ALK performed the statistical analysis. ALK drafted the paper and RJM and JIR modified and refined it. All authors read and approved the final manuscript.
